# Inflammation-induced endothelial cell-derived extracellular vesicles modulate the cellular status of pericytes

**DOI:** 10.1038/srep08505

**Published:** 2015-02-17

**Authors:** Seiji Yamamoto, Shumpei Niida, Erika Azuma, Tsutomu Yanagibashi, Masashi Muramatsu, Ting Ting Huang, Hiroshi Sagara, Sayuri Higaki, Masashi Ikutani, Yoshinori Nagai, Kiyoshi Takatsu, Kenji Miyazaki, Takeru Hamashima, Hisashi Mori, Naoyuki Matsuda, Yoko Ishii, Masakiyo Sasahara

**Affiliations:** 1 Department of Pathology, Graduate School of Medicine and Pharmaceutical Sciences, University of Toyama, Toyama, Japan; 2 Bio Bank Omics Unit, National Center for Geriatrics and Gerontology, Aichi, Japan; 3 Manufacturing & Engineering Lab., Astellas Pharma Inc., Tsukuba, Japan; 4 Toyama Prefectural Institute for Pharmaceutical Research, Toyama, Japan; 5 Department of Cancer Genetics, Roswell Park Cancer Institute, Buffalo, NY, USA; 6 Medical Proteomics Laboratory, Institute of Medical Science, The University of Tokyo, Tokyo, Japan; 7 Department of Immunobiology and Pharmacological Genetics, Graduate School of Medicine and Pharmaceutical Sciences, University of Toyama, Toyama, Japan; 8 The Center for Graduate Medical Education, Jichi Medical University, Tochigi, Japan; 9 Department of Molecular Neuroscience, Graduate School of Medicine and Pharmaceutical Sciences, University of Toyama, Toyama, Japan; 10 Department of Emergency and Critical Care Medicine, Nagoya University, Nagoya, Japan

## Abstract

Emerging lines of evidence have shown that extracellular vesicles (EVs) mediate cell-to-cell communication by exporting encapsulated materials, such as microRNAs (miRNAs), to target cells. Endothelial cell-derived EVs (E-EVs) are upregulated in circulating blood in different pathological conditions; however, the characteristics and the role of these E-EVs are not yet well understood. *In vitro* studies were conducted to determine the role of inflammation-induced E-EVs in the cell-to-cell communication between vascular endothelial cells and pericytes/vSMCs. Stimulation with inflammatory cytokines and endotoxin immediately induced release of shedding type E-EVs from the vascular endothelial cells, and flow cytometry showed that the induction was dose dependent. MiRNA array analyses revealed that group of miRNAs were specifically increased in the inflammation-induced E-EVs. E-EVs added to the culture media of cerebrovascular pericytes were incorporated into the cells. The E-EV-supplemented cells showed highly induced mRNA and protein expression of VEGF-B, which was assumed to be a downstream target of the miRNA that was increased within the E-EVs after inflammatory stimulation. The results suggest that E-EVs mediate inflammation-induced endothelial cell-pericyte/vSMC communication, and the miRNAs encapsulated within the E-EVs may play a role in regulating target cell function. E-EVs may be new therapeutic targets for the treatment of inflammatory diseases.

Cell-to-cell communication is mediated by secreted bioactive molecules, such as short form peptides, proteins, lipids, and nucleic acids. These small molecules are commonly released by cells and bind to specific receptors on target cells, which induces intracellular signaling and changes the target cell's pathophysiological state. Extracellular vesicles (EVs), which include microparticles, microvesicles, and exosomes^1^,^2^,^3^,^4^, are released from different cell types, and emerging evidence suggests that EVs function as carriers of these bioactive molecules^5^,^6^,^7^,^8^.

Clinically, EVs are found in circulating blood, and the number of EVs is elevated in acute and chronic inflammatory diseases, such as sepsis, stroke, preeclampsia, atherosclerosis, diabetes mellitus, and metabolic syndrome^9^,^10^,^11^,^12^,^13^,^14^. Vascular endothelial cells are thought to be one of the major cell types that release EVs into the blood stream^15^. The number of endothelial-derived EVs (E-EVs) circulating in the blood stream correlates with the severity of disease; however, the pathophysiological significance of E-EVs is largely unknown^12^.

MicroRNAs (miRNAs) are small, single-stranded, non-coding RNAs that are transcribed in the nucleus. They are processed by the enzymes Drosha and Dicer, incorporated into RNA-induced silencing complexes, and mediate the translational inhibition or degradation of target mRNAs^16^,^17^. Many miRNAs have been shown to play key roles in pathophysiological processes^18^,^19^. In particular, the inflammation-related miRNAs, miR-101, miR-144, and miR-155, were reported to modulate protein biogenesis in lung epithelial and endothelial cells^20^,^21^. These miRNAs can be carried by E-EVs; however, their roles in E-EV-mediated cell-to-cell communication are not yet known.

Vascular endothelial cells and pericytes/vascular smooth muscle cells (vSMCs) are juxtapositioned to each other in blood vessels^22^. The interactions between these two cell types are important for the regulation of vascular integrity, and perturbation of their interaction has been observed in many diseases, including inflammatory diseases that cause vascular dysfunction, such as disturbance of the blood brain barrier (BBB) in cerebral blood vessels^23^,^24^,^25^,^26^. Here, we aimed to determine the involvement of EVs in cerebrovascular endothelial cell-pericyte communication in inflammatory disease. We found that the E-EVs induced by inflammatory stimuli carry numerous specific miRNAs and can induce pericyte responses to endothelial cells. These results suggest that E-EVs are an important mediator of vascular cell communication in inflammatory conditions.

## Results

### Induction of inflammatory responses in cerebrovascular endothelial cells

**Figure 1:**
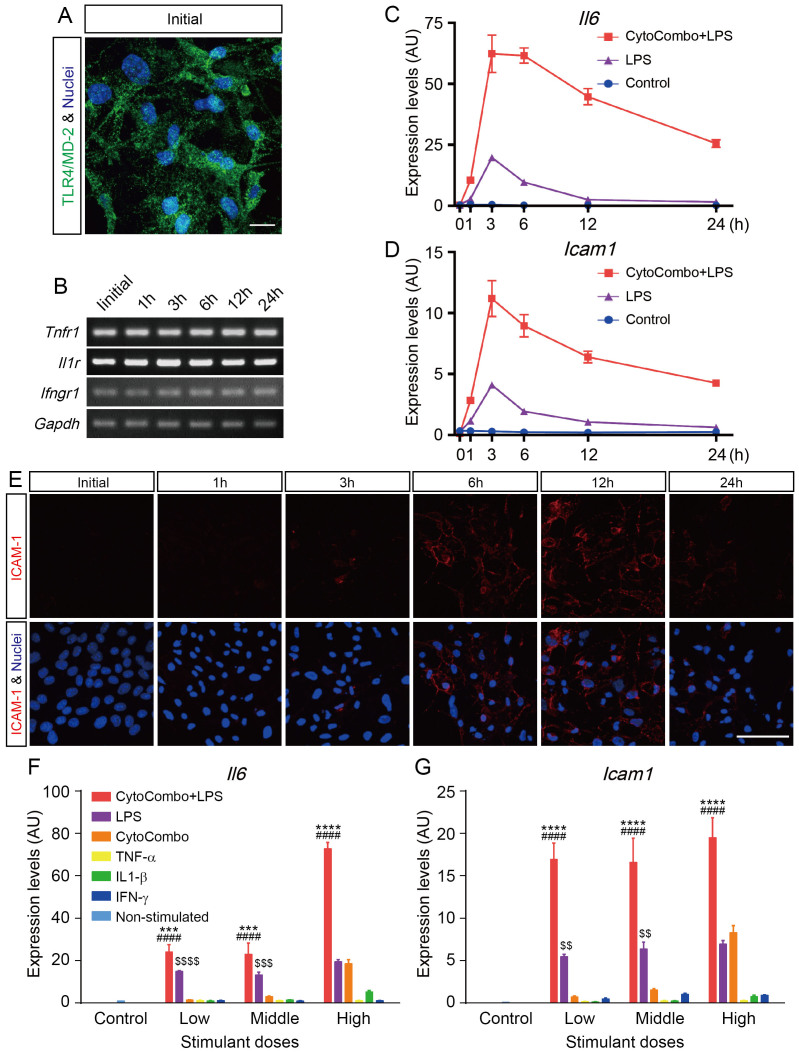
Inflammation-related receptor gene and protein expression levels in cerebrovascular endothelial cells. (a) Expression of the TLR4/MD-2 protein complex was confirmed by immunocytofluorescence (Green). Unstimulated b.End5 cells constitutively express the TLR4/MD-2 protein complex. Nuclei are stained with Hoechst (Blue). Scale bar, 10 μm. (b) Analysis of *Tnfr1*, *Il1r*, and *Ifngr1* expression by conventional RT-PCR. Unstimulated b.End5 cells constitutively express *Tnfr1*, *Il1r*, and *Ifngr1*, and these genes are continuously expressed after 24 hours of stimulation with CytoCombo + LPS. (c and d) Inflammatory gene expression levels in b.End5 cells. *Il6* (c) and *Icam1* (d) are markers of inflammatory responsive genes (n = 4 for each time point). Both genes peaked at 3 hours after stimulation. (e) ICAM-1 protein expression levels were confirmed by immunocytofluorescence. ICAM-1 protein expression levels were well correlated with the *Icam1* mRNA expression pattern. Scale bars, 50 μm. (f and g) Inflammatory gene expression levels in b.End5 cells incubated with various stimulants. The inflammatory responsive genes *Il6* (f) and *Icam1* (g) were measured at 3 hours after stimulation (n = 4 for each time point). Cells stimulated with CytoCombo + LPS showed the most intensive inflammation. Non-stimulated control indicates basal expression level of marker genes. **** P < 0.0001, and *** P < 0.001, CytoCombo + LPS *vs*. LPS; ^####^P < 0.0001, CytoCombo + LPS *vs*. CytoCombo; ^$$$$^P < 0.0001, ^$$$^P < 0.001, and ^$$^P < 0.01, LPS *vs*. CytoCombo.

To analyze the pathobiology of E-EVs released in inflammatory conditions, we developed a reproducible *in vitro* system to induce the production of E-EVs from b.End5 cells, a cerebrovascular endothelial cell line. First, we confirmed that b.End5 cells expressed the LPS receptor TLR4/MD-2 complex under unstimulated conditions by immunocytofluorescence (Fig. 1a). The mRNAs of the inflammatory cytokine receptors *Tnfr1* (for TNF-α), *Il1r* (for IL-1β), and *Ifngr1* (for IFN-γ) were detected in unstimulated b.End5 cells by conventional RT-PCR (Fig. 1b). The gene expression levels were consistent up to 24 hours after stimulation with a high dose of CytoCombo + LPS (a mixture of TNF-α, IL-1β, IFN-γ, and LPS; Supplementary Table 1).

As inflammatory stimuli have been reported to upregulate IL-6 and ICAM-1 expression levels^27^,^28^, we determined the inflammatory responses in b.End5 cells to inflammatory cytokine and endotoxin exposure by measuring *Il6* and *Icam1* gene expression. In real-time PCR analyses, a high dose of CytoCombo + LPS (Supplementary Table 1) induced *Il6* and *Icam1* mRNA expression, which peaked after 3 hours of stimulation (Fig. 1c and d). LPS alone also induced *Il6* and *Icam1* mRNA expression, but at lower levels than CytoCombo + LPS (Fig. 1c and d). In accordance with the *Icam1* mRNA expression (Fig. 1d), the immunoreactivity of ICAM-1 in b.End5 cells was detectable at 3 hours and peaked at 6 to 12 hours after CytoCombo + LPS stimulation (Fig. 1e).

To determine the optimal stimulatory conditions for inducing inflammatory responses in b.End5 cells, the dose effects of the inflammatory stimuli were determined by real-time PCR analyses of *Il6* and *Icam1* mRNA at 3 hours after stimulation. The expression of *Il6* and *Icam1* mRNA was significantly induced by CytoCombo + LPS, CytoCombo (a mixture of TNF-α, IL-1β, and IFN-γ), and LPS (Fig. 1f and g). However, the mRNA levels induced by CytoCombo + LPS were significantly higher than those induced by the other stimuli at all doses examined. The mRNA levels induced by the low and middle doses of LPS were significantly higher than those induced by the low and middle doses of CytoCombo, whereas the high doses of both stimuli induced equivalent mRNA expression levels (Fig. 1f and g). The induction of *Il6* and *Icam1* mRNA was not significant when b.End5 cells were stimulated with either cytokine alone. As an apoptotic cell death triggered by the inflammatory cytokines and LPS^29^,^30^, has been reported to induce the release of apoptotic bodies^4^, we examined apoptosis in b.End5 cells that were exposed to inflammatory stimuli. As compared with non-stimulated control, 3-hour exposure to any kinds of stimulants did not significantly increase the cleaved caspase 3-positive apoptotic cell. Twenty four-hour exposure to the stimulants except for that of low and middle dose of CytoCombo significantly increased cleaved caspase 3-positive apoptotic cell (Supplementary Figure 1a and b). Based on these data, we exposed b.End5 cells to CytoCombo + LPS, LPS, and CytoCombo for 3 hours when induction of apoptosis is still not evident, and then examined the inflammatory simulation-induced E-EV release from these cells.

### Inflammation induced E-EV release from cerebrovascular endothelial cells

**Figure 2:**
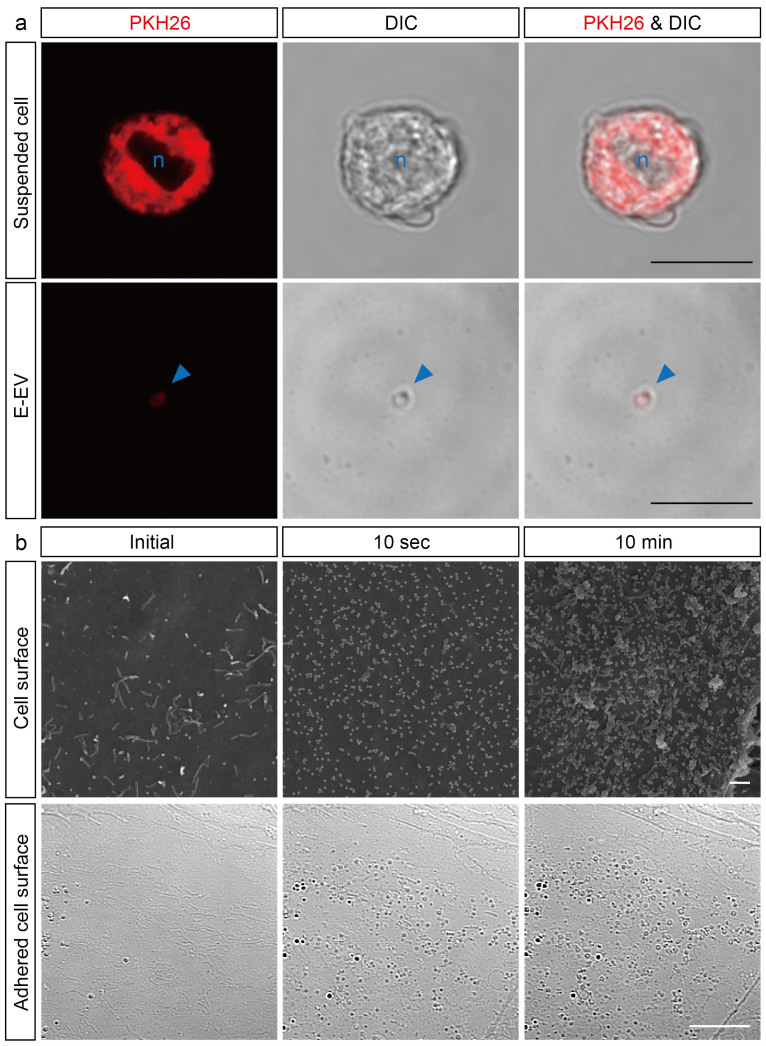
Inflamed cerebrovascular endothelial cells produce shedding-type E-EVs. (a) Treatment of inflamed cells with PKH26, a plasma membrane-intercalating fluorescent reagent, clearly demonstrates that E-EVs are released from the inflamed endothelial cell membranes. Suspended b.End5 cells stained with PKH26 emit red fluorescence (Upper row). Azure-blue n indicates nucleus. PKH26-positive E-EVs are present in the culture medium (Bottom row). Red fluorescence is colocalized to the micron-sized vesicles as observed by differential interference contrast microscopy (DIC). The azure-blue arrowhead shows an E-EV. Scale bar, 10 μm. (b) Scanning electron microscopy (SEM) images showing that E-EVs are shed from the cell surface immediately after stimulation by CytoCombo + LPS (Upper row). The acute response implies that E-EV production is a non-genomic reaction. DIC image showing E-EVs present on the adhered cell surface (Bottom row), suggesting that E-EVs are bilaterally produced on both the luminal and abluminal surfaces of endothelial cells in the tube formed by the blood vessels in inflammatory conditions *in vivo*. Scale bar, 10 μm (a and b lower), and 1 μm (b upper).

We next examined E-EV release from b.End5 cells that were stimulated by CytoCombo + LPS. To identify the E-EVs released from b.End5 cells, the plasma membranes of these cells were prelabeled with PKH26 and these cells were then exposed to the inflammatory stimuli. Three hours after exposure to a high dose of CytoCombo + LPS, PKH26 fluorescence was detected on the plasma membrane of b.End5 cells (Fig. 2a, upper row). Then, we identified PKH26-positive objects of submicron to micron size within the E-EVs fractionated from the culture medium of PKH26-positive b.End5 cells after a 3-hour stimulation with the high dose of CytoCombo + LPS, and confirmed that these fractionated materials were b.End5-derived globules. We further characterized them using the extracellular vesicle markers, such as vascular endothelial growth factor receptor 2 (VEGFR-2) and CD62E (E-selectin)^31^. These globular objects in E-EV fraction were immunoreactive for VEGFR-2 and CD62E under confocal microscopy (Supplementary Figure 2). In addition, immuno-reactive bands of VEGFR-2 and CD62E were enriched in the E-EV fraction than in b.End5 cell lysates by western blot analyses (Supplementary Figure 3).

The surface of the plasma membrane of the inflamed endothelial cells was examined by scanning electron microscopy (SEM). In most of unstimulated b.End5 cells (Initial), we observed few cellular processes like microvilli and limited number of sheddings on the surface of the plasma membrane (Fig. 2b, upper), whereas small fraction of unstimulated cells (2.38 ± 5.83%) showed similar changes as seen in those with stimulation. At 10 seconds after the addition of high dose CytoCombo + LPS to the culture medium, many sheddings were emerged on the surface of plasma membrane, and the microvilli turned to be obscure in all stimulated cells (100%; Fig. 2b, upper row). Higher magnification images further revealed the tiny and dome-shaped swellings (less than 0.1 μm in diameter) on the surface of plasma membrane, which structure may correspond to the early phase of sheddings (Supplementary Figure 4a). The sheddings were further increased at 10 minutes, and most of them ranged between 0.1 μm and 0.4 μm in diameter (Fig. 2b, upper row). At higher magnification, sheddings of larger-size (≥0.3 μm) and those of aggregated form (up to 3 μm) were increased as compared with those at 10-second of stimulation (Supplementary Figure 4b). In addition, similar shedding-like structures were observed on the bottom surface of the cells that were exposed to high-dose CytoCombo + LPS by using the interference contrast microscopy (DIC) mode of a confocal microscope (Fig. 2b, bottom row). These data strongly suggest that E-EV production is mainly membrane-shedding type^15^,^32^ and may be partly from multivesicular bodies (MVBs)^33^, and the plasma membrane shedding of E-EVs induced by inflammatory stimulants is very acute. In addition, our findings may support the possibility that E-EVs could be produced bilaterally, on both the luminal and abluminal surfaces, from inflammation-stimulated endothelial cells in cerebral blood vessels *in vivo*.

**Figure 3:**
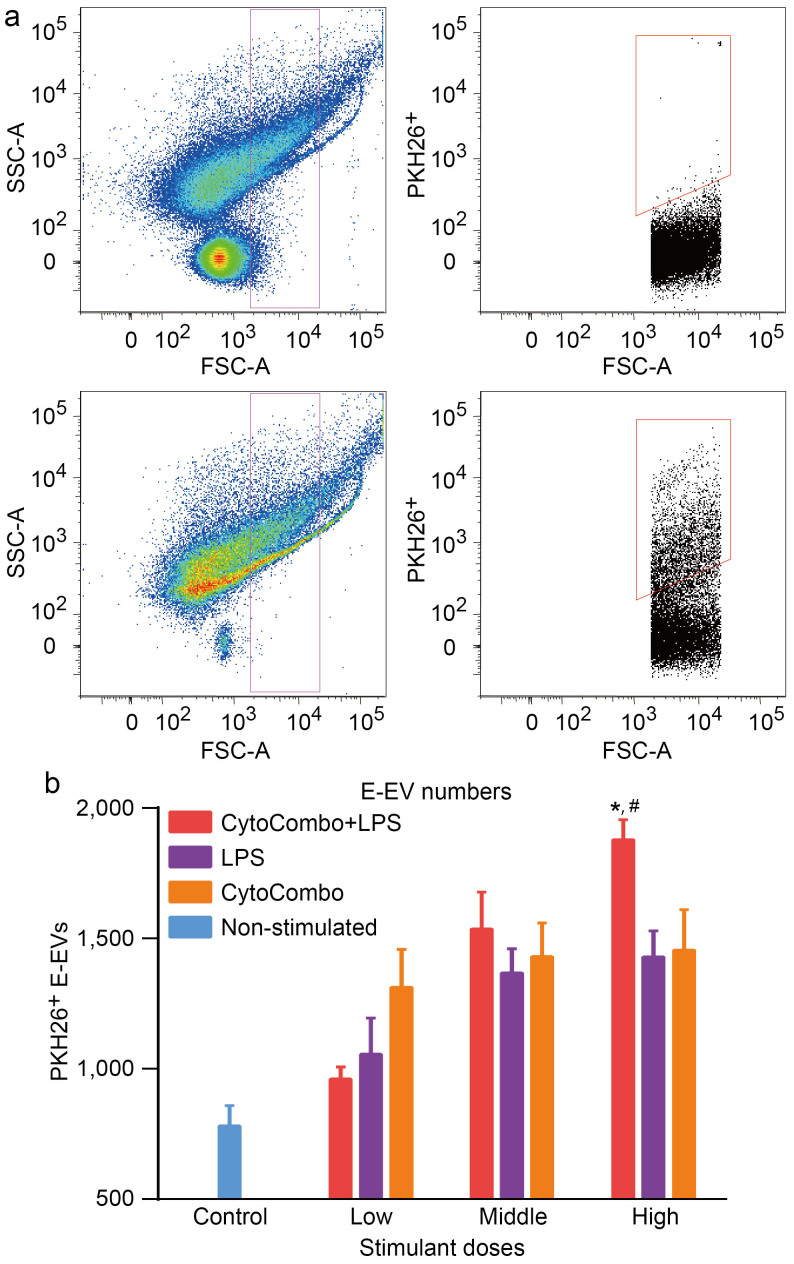
Quantitative analysis of E-EVs by flow cytometry. (a) PKH26-positive E-EVs were detected and measured quantitatively by flow cytometry. The upper graphs show the pre-culture medium with CytoCombo + LPS. The lower graphs show the culture medium from b.End5 cells stained with PKH26 stimulated by CytoCombo + LPS for 3 hours. Vesicles gated from submicron to micron size (0.3 μm to 3.0 μm in diameter, magenta) are shown in the graph on the left. In the graphs on the right, PKH26-positive E-EVs are gated (red). The pre-culture medium contains many FBS-derived vesicles that are negative for PKH26. E-EVs can be observed in the 3-hour culture medium as the PKH26-positive fraction. (b) E-EV production is significantly upregulated after 3 hours of exposure to the high dose of CytoCombo + LPS. In contrast, a lower number of E-EVs are observed in the LPS and CytoCombo groups (reported as the number of PKH26-positive E-EVs in 100 μL of medium, n = 4 for each). Non-stimulated control indicates basal production level of E-EVs. * P < 0.05, CytoCombo + LPS *vs*. LPS; ^#^P < 0.05, CytoCombo + LPS *vs*. CytoCombo.

Next, E-EV release from b.End5 cells prelabeled with PKH26 was quantitatively analyzed by flow cytometry. Based on the above mentioned SEM study, we focused on particles between 0.3 μm and 3.0 μm in diameter, which corresponds to the diameter of the E-EVs released in membrane-shedding mode^32^,^34^,^35^. The preculture medium containing CytoCombo + LPS included many particles between 0.3 μm and 3.0 μm in diameter that were negative for PKH26, such as FBS-derived EVs, and EVs from MVBs (Fig. 3a, upper graphs). In contrast, after 3 hours of culture with high dose CytoCombo + LPS, the culture medium contained a significant number of PKH26-positive particles between 0.3 μm and 3.0 μm in diameter (Fig. 3a, bottom graphs). These findings indicated that the plasma membrane-intercalating nature of PKH26, with which the b.End5 cells were prelabeled, allowed us to discriminate foreign substances in the medium from E-EVs and to precisely measure the release of E-EVs. In a comparative assay, the three types of stimulants significantly induced the release of PKH26-positive E-EVs compared to that released from the non-stimulated control (stimulant, P-value *vs.* the control; CytoCombo + LPS, P < 0.0001; CytoCombo, P < 0.01; LPS, P < 0.05; Fig. 3b). CytoCombo + LPS induced the release of E-EVs in a dose-dependent manner up to the high dose, and the release induced by CytoCombo + LPS was significantly higher than that induced by the other stimuli at the high dose (Fig. 3b). At the high doses, CytoCombo and LPS individually induced the release of E-EVs to similar extents. These changes of E-EV production were well correlated with those of *Il6* and *Icam1* mRNA induction, both of which were induced by different inflammatory stimuli. Accordingly, these data suggested that E-EV production was correlated with the level of cellular inflammation. At the low and middle doses, CytoCombo induced E-EV release (P < 0.01 and P < 0.001, respectively, *vs*. the control) but not *Il6* and *Icam1* mRNA (both n.s. *vs.* the control; Fig. 1f and g). Thus, E-EV production in cerebrovascular endothelial cells appears to be a more sensitive cellular response than cytokine production based on relative induction by inflammatory stimulants.

### Inflammation-induced E-EVs may target cerebrovascular pericytes

**Figure 4:**
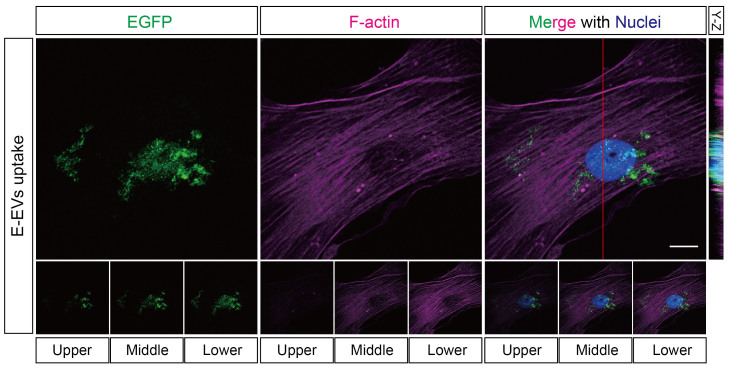
E-EVs are incorporated into cerebrovascular pericytes. Plasma membrane-localized EGFP (M-EGFP)-positive E-EVs were fractionated from M-EGFP-expressing b.End5 cells. When the M-EGFP-positive E-EVs were incubated with cerebrovascular pericytes, the E-EVs were taken up by the cells as confirmed by immunocytofluorescence of EGFP. Incorporation of the E-EVs by the cells was precisely confirmed by a Y-Z cross-sectional image (adjacent to the 3D projection merged image) and by 1-μm, sequential, single Z-plane images (bottom row). Scale bar, 10 μm.

Endothelial cells and pericytes are juxtapositioned and functionally correlated in the vasculature^22^,^36^. Next, we determined if the miRNAs in E-EVs could mediate the inflammatory signaling between cerebrovascular endothelial cells and pericytes. To this end, we first assessed whether E-EVs could be incorporated into pericytes. To rule out the effect of modification to the E-EV surface by the plasma membrane-intercalating dye PKH26, we constructed a membrane-localized EGFP (M-EGFP)-coding vector. M-EGFP-positive E-EVs were fractionated from the culture medium of M-EGFP expressing b.End5 cells after a 3-hour stimulation with the high dose of CytoCombo + LPS. The E-EV fractions obtained were washed 3 times with PBS, and then added to the culture medium of human brain pericytes (HBPCs). After a 24-hour incubation, immunocytofluorescence showed that many M-EGFP-positive E-EVs were incorporated into the cytoplasmic region of HBPCs (Fig. 4). Some of the M-EGFP-positive E-EVs were localized near the nucleus, as confirmed by the Y-Z cross-sectional image and single-plane sequential Z images (Fig. 4, upper right and bottom row). These findings suggest that the bioactive molecules encapsulated in the E-EVs were transferred into HBPCs.

**Figure 5:**
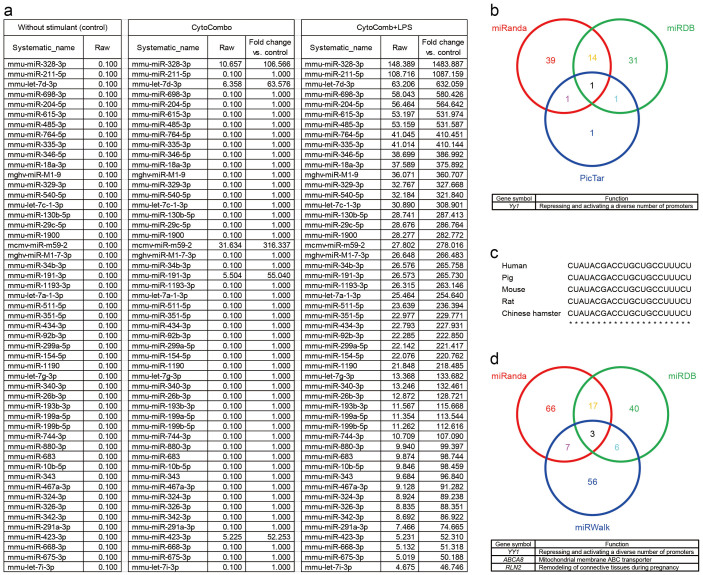
MiRNA array data from inflammation-stimulated E-EVs. (a) MiRNAs extracted from E-EVs undergo a drastic change following treatment with inflammatory stimulants. Some of the miRNAs are upregulated more than 500 fold by CytoCombo + LPS treatment when compared to the non-stimulated control. (b) The multifunctional transcription factor *Yy1* is determined as a target of let-7d-3p in mice that are commonly identified in 3 different public accessible databases. (c) Homology analysis of let-7d-3p. The alignment of let-7d-3p sequences indicates that the mature sequences of five mammalian species are identical. (d) *YY1, ABCA8,* and *RLN2* are determined as a target of let-7d-3p in human that are commonly identified in 3 different public accessible databases.

To examine the bioactive molecules encapsulated in the E-EVs, we examined the molecules in the E-EV fractions from b.End5 cells with an miRNA array. Many miRNAs were upregulated in the E-EVs from cells stimulated by a high dose of CytoCombo + LPS compared to those in the E-EVs from non-stimulated control cells (Fig. 5a). Some of them were also upregulated in the E-EVs from cells stimulated with a high dose of CytoCombo, which induced an inflammatory response in b.End5 cells, although to a lesser extent than CytoCombo + LPS (Fig. 5a). These miRNAs sorted into the E-EVs were classified by Ingenuity Pathway Analysis (IPA). A significant proportion of the miRNAs were approved to be related to Inflammatory Disease and Inflammatory Response (bright red) in Disease and Disorders of the IPA (Supplementary Figure 5). Moreover, the miRNAs were abundant in those related to Organismal Injury and Abnormalities, which may associate with inflammation, over-represented (dark red). These data suggest that inflammatory stimuli are able to sort the inflammation-related miRNAs into E-EVs.

**Figure 6:**
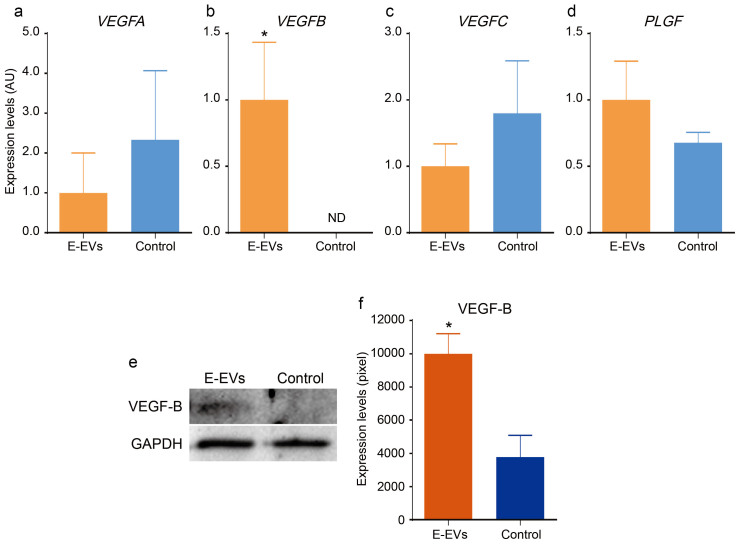
Effects of E-EVs in cerebrovascular pericytes. (a–d) The mRNA levels of the VEGF family members, *VEGFA*, *VEGFC*, and *PLGF* in the E-EV-supplemented and non-stimulated control groups did not differ significantly (a, c, and d). In contrast, *VEGFB* was significantly upregulated in the E-EV-supplemented group compared to the non-stimulated control group (b) (n = 3, P < 0.05). (e and f) VEGF-B protein levels were measured by western blotting (e), using GAPDH as an internal control. VEGF-B protein was significantly upregulated in the E-EV-supplemented group compared to the non-stimulated control group (f) (n = 3, P < 0.05).

Among the various inflammation-related miRNAs detected (Fig. 5a), we focused on two, miR-328-3p and let-7d-3p, because these miRNAs were extensively induced after stimulation with CytoCombo + LPS, and were significantly induced, but to a lesser extent, after CytoCombo stimulation. Real-time PCR showed that the cultured HBPCs expressed *Yin and yang 1* (*YY1*), which is a target mRNA of let-7d-3p and is an orthologue of mouse *Yy1* that was identified through in silico analysis (Figure 5b), but not the target mRNAs of miR-328-3p including *Proviral integration site for Moloney murine leukemia virus 1* (*PIM1*), *Testis-specific kinase 2* (*TESK2*), and *Ataxin-2 binding protein 1* (*A2BP1*) (data not shown). The well-conserved, mature let-7d-3p sequences in mice and humans are identical (Fig. 5c) and were shown to target *YY1* mRNA through *in silico* analyses (Fig. 5d). VEGF family proteins are synthesized in pericytes/vSMCs^37^,^38^ and are believed to be the downstream targets of *YY1*, since *YY1* knockdown upregulates their expression levels, in which the upregulation of VEGF-B was most striking among VEGF family proteins^39^. Therefore, we investigated whether inflammation-induced E-EVs modulated the expression of VEGF family mRNAs and proteins. We observed that VEGF-B mRNA and protein levels were low in the controls, but were extensively induced in the E-EV-supplemented group (Fig. 6b, e, f). In contrast, E-EV supplementation did not affect *VEGFA*, *VEGFC*, or *PLGF* mRNA levels (Fig. 6a, c, d). The miRNAs carried by E-EVs may mediate the inflammatory responses of endothelial cells by regulating gene expression profiles in pericytes/vSMCs, especially in cerebrovascular pericytes.

## Discussion

Emerging evidence has suggested the importance of EVs in cell-to-cell communication as carriers of small molecules such as peptides, proteins, lipids, mRNAs, and miRNAs^40^,^41^. The bioactive molecules encapsulated in EVs can be transferred to and function in target recipient cells^6^,^42^,^43^,^44^. In addition, miRNAs act as regulatory molecules of diverse biological phenomena, such as immune responses, inflammation, and tumor growth^18^,^19^. However, our knowledge of EV- and encapsulated miRNA-mediated cell-to-cell communication is mainly limited to tumor cell biology. Although it is known that the proinflammatory agent TNF-α induces the release of E-EVs^45^, the significance of these E-EVs remains to be elucidated. To the best of our knowledge, we demonstrated here, for the first time, that the inflammation-related miRNAs carried by the E-EVs from endothelial cells mediated inflammatory responses in pericytes. The induction of E-EV shedding was very acute and sensitive compared to previously established inflammation markers like IL-6 and ICAM-1^27^,^28^, and it may be useful as a very sensitive marker for inflammatory diseases.

In recent years, a variety of pathogens and disease states have been shown to affect the composition and function of these EVs. *In vitro* studies demonstrated that environmental stresses such as heat, hypoxia, irradiation, and changing of conditioned medium, differentially modified the composition of EVs^46^,^47^,^48^,^49^. Specific coding and non-coding RNAs, retrotransposon RNAs, Alu transposable elements, and chromosomal and mitochondrial DNA have been reported to be enriched in tumor cell-derived EVs^50^,^51^,^52^,^53^,^54^. The results of our present study showed that when exposed to inflammatory stimuli, cerebrovascular endothelial cells could sort many inflammation-related miRNAs into E-EVs *ad initium*. Moreover, the type and amount of miRNAs that were sorted into the E-EVs was strongly correlated with the severity of endothelial inflammation.

EVs from tumor cells could educate the bone marrow progenitor cells to mobilize out from their niche^55^. Vascular endothelial cells in tumors could be “educated” by EVs derived from tumor cells to exhibit a highly proliferative phenotype and karyotype abnormalities^7^,^56^. Along these same lines, our results show that the molecules in the inflammation-induced E-EVs function in target pericytes to increase VEGF-B mRNA and protein expression. Furthermore, this “education” was suggested to be mediated by the miRNA encapsulated in the E-EVs since *VEGFB* is thought to be a downstream target of the highly-accumulated miRNA in the inflammation-induced E-EVs. Similarly, various organ-derived EVs have been shown to be incorporated into bone marrow cells *in vitro*^57^. As a result, the mRNAs were delivered and the expression of organ-specific mRNAs was induced in the bone marrow cells. E-EVs and encapsulated miRNA were shown to be involved in the cascade of cellular events in the inflammatory vascular response.

VEGF-B is a specific ligand of VEGFR-1 that is expressed in endothelial cells and pericytes/vSMCs^58^,^59^. VEGFR-1 mediates pathological angiogenesis by targeting both endothelial cells and pericytes in the pathological neovascularization of choroidal and retinal tissues^60^. Similarly, VEGFR-1 stimulates the endothelial differentiation and the formation of blood vessels in infantile hemangioma^61^ and vascular formation in implanted tumors^62^. Furthermore, VEGFR-1 ablated vascular pericytes and aggravated vascular leakage to induce cancer-associated retinopathy^63^. Accordingly, inflammation-induced E-EVs enhanced VEGF-B expression in pericytes, which may mediate pathological neovascularization and/or vascular leakage in response to inflammation of endothelial cells. Furthermore, since E-EVs are carried in the blood stream in many pathological conditions, E-EVs may spread the local inflammatory responses of endothelial cells to the systemic vasculature. Inhibition of E-EVs may limit the extent of inflammation-induced cellular responses from endothelial cells to the surrounding cells, and E-EVs may be a new therapeutic target for inflammatory diseases.

In conclusion, we developed a reproducible *in vitro* E-EV production system and a quantitative E-EV measurement method. Using these tools, we demonstrated that inflammation-induced E-EVs could “educate” target cells to modulate growth factor production. We also showed evidence to suggest that part of this cell education signal might be an miRNA encapsulated in the E-EVs. Our findings offer a new clinical approach, targeting E-EVs, for the prevention and treatment of acute and chronic inflammatory diseases. Further experiments are needed to elucidate the molecular mechanisms by which E-EVs transfer their “educating” messages.

## Methods

### Cell lines and culture conditions

The mouse brain endothelial cell line, b.End5, and human brain pericytes (HBPCs) were purchased from DS Pharma Biomedical (Osaka, Japan), and from Applied Cell Biology Research Institute (Kirkland, WA), respectively. The cells were cultured using the EBM-2 bullet kit (Takara, Kyoto, Japan) at 37°C in a humidified atmosphere containing 5% CO_2_. Membrane-localized EGFP (M-EGFP)-expressing b.End5 cells were generated by transfection of the *GAP43* *membrane targeting sequence-Egfp* fusion gene, which was constructed as follows. Briefly, the human *GAP43 membrane targeting sequence* was cloned from the plasmid (Clone ID No. IRAL020F10, RIKEN BRC, Tsukuba, Japan) and inserted into the multi-cloning site of the pEGFP-N1 vector (Takara). The vector containing the resultant fusion gene, consisting of the *GAP43* *membrane targeting sequence* and *Egfp*, was transfected into b.End5 cells using X-tremeGENE transfection reagent (Roche, Basel, Switzerland) according to the manufacturer's protocol.

### Reagents

The inflammatory cytokines TNF-α, IL-1β, and IFN-γ were purchased from PeproTech (Rocky Hill, NJ). Lipopolysaccharide (LPS) was obtained from List Biological Laboratories (Campbell, CA). TNF-α, IL-1β, and IFN-γ were used separately, as a mixture (referred to as CytoCombo), or as a mixture of CytoCombo and LPS (CytoCombo + LPS) (Supplementary Table 1). The plasma membrane intercalating fluorescent dye PKH26 was purchased from Sigma-Aldrich (St. Louis, MO) and was used according to the manufacturer's protocol. The cell tracer fluorescence dye 5-(and 6)-carboxyfluorescein diacetate succinimidyl ester (CSFE) was purchased from eBioscience (San Diego, CA) and was used according to the manufacturer's protocol.

### Fractionation of endothelial cell-derived extracellular vesicles (E-EVs)

Ten-centimeter dishes coated with type I collagen (BD Biosciences, San Jose, CA) of confluent b.End5 cells were rinsed with PBS, and stimulated with medium containing CytoCombo + LPS, CytoCombo, or LPS alone to induce inflammation. Culture medium without any stimulant was used as a control. After a 3-hour incubation, the conditioned culture medium was centrifuged at 1,000 rpm for 10 minutes at 4°C to eliminate the cellular debris. The supernatant was then ultracentrifuged at 100,000 × *g* for 90 minutes at 4°C, and the pellets were used as the E-EV fraction in the following analyses.

### Immunocytofluorescence

For immunocytofluorescence, cells were seeded into 8-well culture slides (BD Biosciences). After treatment with various stimuli, the slides were fixed in 4% paraformaldehyde for 15 minutes on ice, and then rinsed with PBS. All specimens were permeabilized in a 0.3% Triton X-100/PBS solution. Permeabilized specimens were incubated at 4°C overnight with primary antibodies diluted in 0.03% Triton X-100/PBS with 10% normal goat serum (VECTOR LABORATORIES, Burlingame, CA), which was used for blocking. The following primary antibodies were used: rat anti-TLR4/MD-2 complex (1:200; eBioscience), mouse anti-ICAM-1 (1:200; Abcam, Cambridge, UK), rabbit anti-cleaved caspase 3 (1:200; Cell Signaling Technology, Danvers, MA), rabbit anti-GFP (1:100; Frontier Institute, Hokkaido, Japan). The Alexa-Fluor488, Alexa-Fluor568, or Alexa-Fluor633-conjugated secondary antibodies (Life Technologies Corporation, Carlsbad, CA) were used at 1:500 dilutions. For F-actin staining, Falloidin-Alexa-Fluor647 (1:500; Cytoskeleton, Denver, CO) was used. Nuclei were stained with Hoechst 33258 (Nacalai Tesque, Koto, Japan). For immunofluorescence of E-EVs, fractionated E-EVs from culture media of b.End5 cells pre-stained by PKH26 or CSFE were incubated with 3% normal mouse serum (VECTOR LABORATORIES) at 4°C for blocking. The following primary antibodies were used: rat anti-Flk-1-FITC (1:20; BD Biosciences, San Jose, CA), rat anti-CD62E-PE (1:20; BD Biosciences). The imaging system was a Leica TCS SP5 confocal system (Leica Microsystems, Wetzlar, Germany).

### FACS analysis

E-EVs released into culture media were analyzed by flow cytometry. E-EVs in the media were gated as events of 0.3 to 3.0 μm in diameter, corresponding to the size of the membrane shed EVs^32^,^33^,^34^. E-EVs were further gated as PKH26-positive events, by using b.End5 cells pre-labeled with PKH26, a membrane intercalating dye. This gating allowed us to discriminate E-EVs from the other microparticles of similar diameters in the medium. We used 3.0- to 3.4-μm rainbow calibration particles (BD Biosciences) as benchmarks. Flow cytometry was performed with a FACSCanto II (BD Biosciences) and FlowJo software (Tree Star, Ashland, OR) was used for data analysis.

### Scanning electron microscopy

Endothelial b.End5 cells were seeded on cover slips and cultured until confluent. Confluent cells were then exposed to inflammatory stimuli (CytoCombo + LPS) for 10 seconds or 10 minutes. After stimulation, the cells were fixed with 2% formaldehyde (Wako Pure Chemical Industries, Osaka, Japan) and 2.5% glutaraldehyde (Wako Pure Chemical Industries) prepared in 0.1 M sodium phosphate buffer (pH 7.4) for 2 hours at room temperature, and then postfixed in 1% osmium tetroxide in the same buffer for 2 hours on ice. Cells were then dehydrated in a graded series of ethanol, substituted with t-butyl alcohol and freeze-dried. Cells were sputter-coated with osmium and then observed in a S4200 scanning electron microscope (Hitachi).

### RNA extraction, cDNA synthesis, conventional PCR, and real-time PCR

Total RNA was isolated from b.End5 cells or HBPCs using the RNeasy Mini Kit (Qiagen, Hilden, Germany) according to the manufacturer's instructions. Total RNA (100 ng) was reverse-transcribed as described in the PrimeScript RT reagent Kit protocol (Takara). Conventional PCR was performed with a Bio-Rad C100 Thermal Cycler (Bio-Rad Laboratories, Hercules, CA). The cDNAs were diluted 1:20 with the reaction mixture from the Ex Taq PCR kit (Takara), and the PCR was performed by standard 3 step amplification protocol consisting of hot start enzyme activation at 95°C for 10 minutes followed by 35 cycles of amplification at 95°C for 30 seconds, 60°C for 60 seconds, and 72°C for 60 seconds. For real-time PCR, which was performed with a Takara Thermal Cycler Dice Real Time System TP800 (Takara), the cDNAs were diluted 1:25 in the reaction mixture containing SYBR Premix EX Taq II (Takara). The real-time PCR program consisted of hot start enzyme activation at 95°C for 10 seconds followed by 45 cycles of amplification at 95°C for 10 seconds and 60°C for 40 seconds. Finally, to obtain a dissociation curve, a final cycle was performed at 95°C for 1 minute, 60°C for 30 seconds, and 95°C for 10 seconds. For data analysis, the mouse or human *glyceraldehyde-3-phosphate dehydrogenase* (*Gapdh* or *GAPDH*) housekeeping gene was used as an internal control. Induction values were calculated using analysis software (Takara). Primer sequences are available upon request from the TAKARA BIO INC. website (http://www.takara-bio.co.jp/).

### MiRNA array

The miRNA expression profile was obtained by using a Mouse miRNA microarray 8 × 15 K (Agilent Technologies, Santa Clara, CA) according to the manufacturer's protocol. Total RNA was prepared from fractionated E-EVs using the miRNA easy Mini Kit (Qiagen). RNA quality was measured with an Agilent 2100 Bioanalyzer (Agilent Technologies). Synthesis of probes, and hybridization to the Mouse miRNA microarray 8 × 15 K were performed by using the miRNA Complete Labeling and Hyb Kit (Agilent Technologies). Hybridized samples were analyzed using a Microarray Scanner (G2505B, Agilent Technologies) and GeneSpring GX software (Agilent Technologies) according to standard protocols. The miRNA array data were analyzed using Ingenuity Pathway Analysis (Ingenuity Systems, http://www.ingenuity.com/).

### Western blotting

For E-EV marker analyses, E-EVs that were fractionated from the culture media of b.End5 cells after CytoCombo + LPS stimulation were used as sample. For the control, b.End5 cells were used. For VEGF-B analyses, E-EVs that were fractionated from the culture media of b.End5 cells after CytoCombo stimulation were added (100 μg) to HBPCs cultured in a collagen type I-coated 24-well plate (BD Bioscience), and the plate was incubated at 37°C for 48 hours. Sample preparations and all other procedures for western blotting have been described elsewhere^64^. Briefly, cells were lysed using RIPA buffer (Thermo Fisher, Waltham, MA) on ice. Whole cell lysates were separated by SDS-PAGE and then electrophoretically transferred to polyvinylidene difluoride membranes. The membranes were incubated for 1 hour at room temperature in a 50 mM Tris buffer containing 150 mM NaCl, 0.05% Tween 20, and 5% BSA for blocking. The membranes were then probed with rabbit anti-VEGFR-2 (1:500; Cell Signaling Technology), rabbit anti-CD62E (1:100; Abcam), rabbit anti-VEGF-B (1:100; Abcam), and mouse anti-GAPDH (1:5,000; Merck Millipore, Billerica, MA) antibodies. The membranes were washed in a 50 mM Tris buffer containing 150 mM NaCl, and 0.05% Tween 20, and were incubated with the appropriate horseradish peroxidase-conjugated secondary antibodies. Immunoreactive bands were detected using enhanced chemiluminescence reagents (GE Healthcare Bio-Sciences AB, Uppsala, Sweden) according to the manufacturer's instructions.

### Statistical analysis

Statistical significance was determined using Student's t-test, or one- or two-way analysis of variance (ANOVA) with Tukey's or Newman-Keuls multiple comparisons as a post hoc analysis for ANOVA. P values less than 0.05 were considered statistically significant. Graphs were drawn using GraphPad Prism 6 (GraphPad Software, Inc., La Jolla, CA). Quantified data are presented as mean ± SEM.

## Author Contributions

S.Y. and S.N. conceived and designed the project. S.Y., E.A., T.Y., M.M., T.-T.H., H.S., S.H., M.I., Y.N., K.M. and H.M. performed experiments. S.Y. wrote the manuscript with significant input from K.T., T.H., Y.I., H.M., N.M., S.N. and M.S. All authors reviewed the manuscript.

## Supplementary Material

Supplementary Information Supplementary data
