# Supplementary material for: Inflammation-induced endothelial cell-derived extracellular vesicles modulate the cellular status of pericytes
Source: Sci Rep. 2015 Feb 17;5:8505. doi: 10.1038/srep08505 (PMC4330530; doi:10.1038/srep08505)
Supplement: Supplementary Information — Supplementary data [file srep08505-s1.pdf]

Inflammation-induced endothelial cell-derived extracellular vesicles modulate the cellular status of pericytes

Seiji Yamamoto[1,*,#], Shumpei Niida[2,#], Erika Azuma[1,3], Tsutomu Yanagibashi[4], Masashi Muramatsu[2,5], Huang Ting Ting[1], Hiroshi Sagara[6], Sayuri Higaki[2], Masashi Ikutani[7], Yoshinori Nagai[7], Kiyoshi Takatsu[4], Kenji Miyazaki[8], Takeru Hamashima[1], Hisashi Mori[9], Naoyuki Matsuda[10], Yoko Ishii[1], Masakiyo Sasahara[1]

[1]Department of Pathology, Graduate School of Medicine and Pharmaceutical Sciences, University of Toyama, Toyama, Japan
[2]Bio Bank Omics Unit, National Center for Geriatrics and Gerontology, Aichi, Japan
[3]Manufacturing & Engineering Lab., Astellas Pharma Inc., Tsukuba, Japan
[4]Toyama Prefectural Institute for Pharmaceutical Research, Toyama, Japan
[5]Department of Cancer Genetics, Roswell Park Cancer Institute, Buffalo, NY, USA
[6]Medical Proteomics Laboratory, Institute of Medical Science, University of Tokyo, Tokyo, Japan
[7]Department of Immunobiology and Pharmacological Genetics, Graduate School of Medicine and Pharmaceutical Sciences, University of Toyama, Toyama, Japan
[8]Jichi Medical University, Tochigi, Japan
[9]Department of Molecular Neuroscience, Graduate School of Medicine and Pharmaceutical Sciences, University of Toyama, Toyama, Japan
[10]Department of Emergency and Critical Care Medicine, Nagoya University, Nagoya, Japan

*Correspondence to: Seiji Yamamoto, Ph.D., Department of Pathology, Graduate School of Medicine and Pharmaceutical Sciences, University of Toyama, Toyama 930-0152, Japan
Tel: +81-076-434-2281, Fax: +81-076-434-5016, E-mail: seiyama@med.u-toyama.ac.jp

[#]These authors contributed equally to this work.

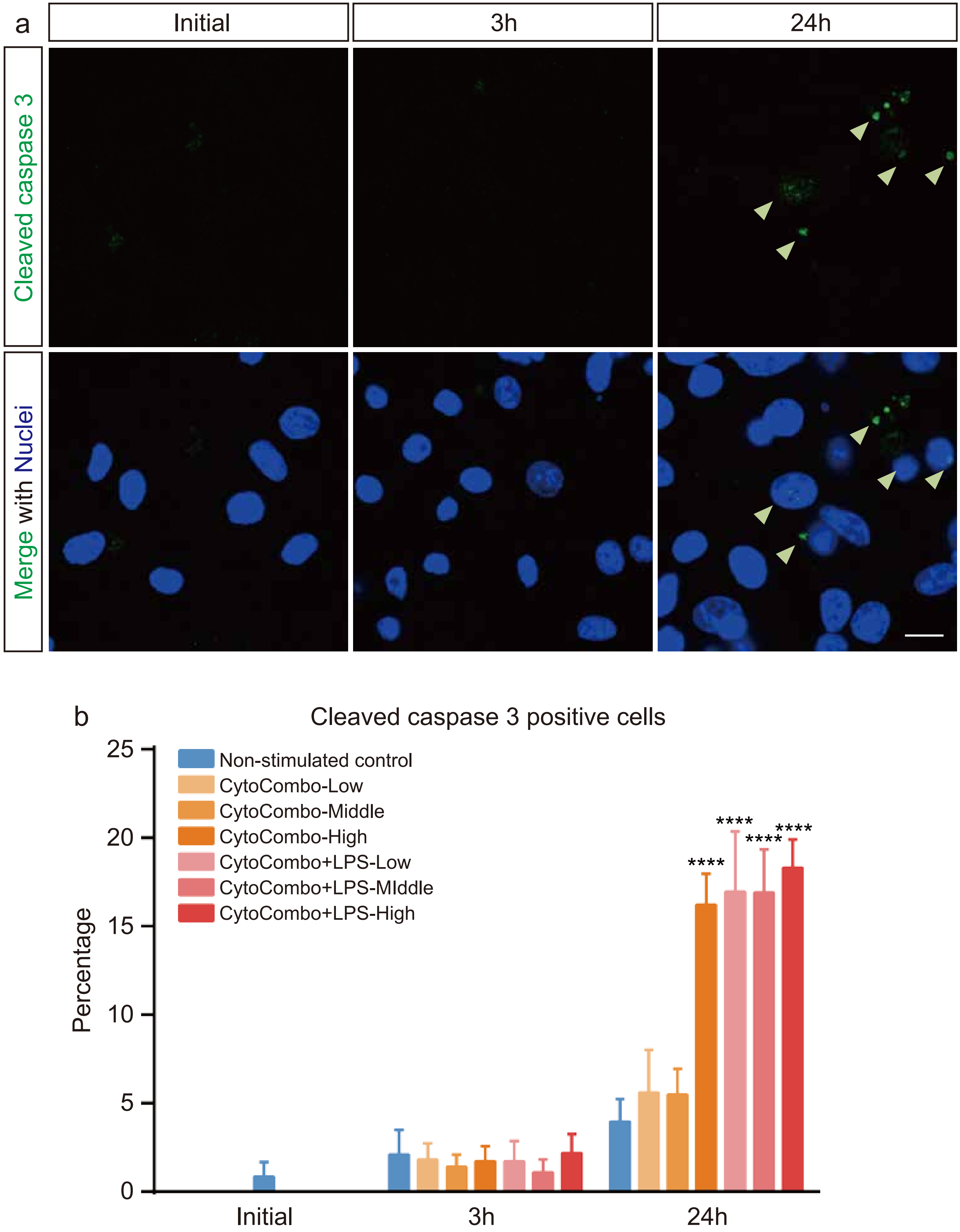

a
Initial
3h
24h
Cleaved caspase 3
Merge with Nuclei
b
Cleaved caspase 3 positive cells
Percentage
25
20
15
10
5
0
Non-stimulated control
CytoCombo-Low
CytoCombo-Middle
CytoCombo-High
CytoCombo+LPS-Low
CytoCombo+LPS-MIddle
CytoCombo+LPS-High
****
****
****
****
Initial
3h
24h

Supplementary Figure 1 | Immunocytofluorescence analysis of apoptosis of b.End5 cells stimulated by CytoCombo+LPS. (a) Representative images of the apoptotic cells in high dose CytoCombo+LPS. b.End5 cells stimulated with CytoCombo+LPS were subjected to immunocytofluorescence at the indicated time points. Three hours after inflammatory stimulation, the number of cleaved caspase 3-positive apoptotic cells was similar to that of initial state. In contrast, the number of cleaved caspase 3-positive apoptotic cells was higher at 24 hours than at the other time points. Scale bar, 10 µm. (b) Quantitative analysis of the apoptotic cells in various concentration of the CytoCombo and CytoCombo+LPS ($n = 8$ for each time point). High dose of CytoCombo and all doses of CytoCombo+LPS at 24 hours significantly increased apoptotic cell death with cleaved caspase 3 immuno-positivity. All different stimulati at 3 hours and CytoCombo of low and middle doses at 24 hours did not significantly increase apoptotic cells as compared to non-stimulated control of "Initial". **** $P < 0.0001$, *vs.* non-stimulated control.

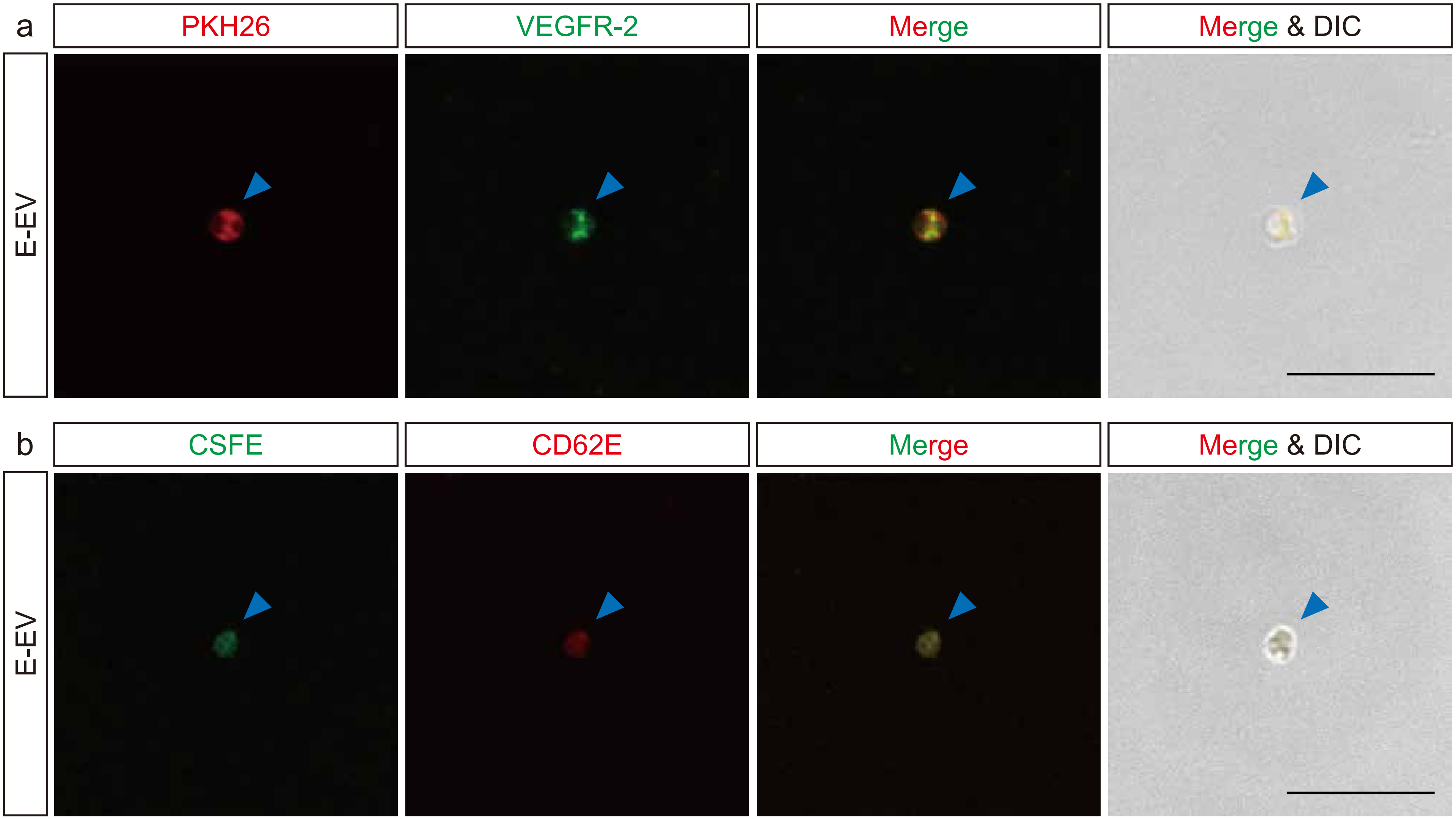
a
PKH26
VEGFR-2
Merge
Merge & DIC
E-EV
b
CSFE
CD62E
Merge
Merge & DIC
E-EV

Supplementary Figure 2 | E-EVs derived from b.End5 cells express extracellular vesicle markers. (a) PKH26-positive E-EVs (red) fractionated from culture supernatant of PKH26 prestained b.End5 cells that were stimulated with high dose of CytoCombo+LPS for 3 hours. PKH26 positive E-EVs express VEGFR-2 as confirmed by immunostaining (green). Red and green fluorescence are colocalized to the micron-sized vesicles as observed by DIC. The azure-blue arrowhead indicates an E-EV. (b) CSFE-positive E-EVs (green) fractionated from culture sup of CSFE prestained b.End5 cells that were stimulated with high dose of CytoCombo+LPS for 3 hours. CSFE positive E-EVs express CD62E as confirmed by immunostaining (red). Green and red fluorescence are colocalized to the micron-sized vesicles as observed by DIC. The azure-blue arrowhead indicates an E-EV. Scale bars, 10 μm.

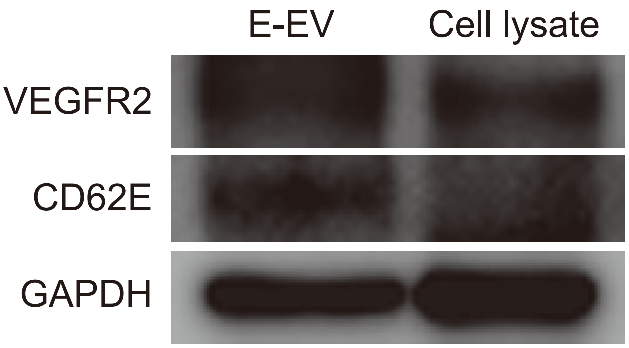
E-EV
Cell lysate
VEGFR2
CD62E
GAPDH

Supplementary Figure 3 | Molecular based characterization of E-EVs. Fractionated E-EVs subjected western blot analysis. Extracellular vesicle markers including VEGFR-2 and CD62E immuno-reactive bands can be observed in E-EV fraction. Cell lysate of b.End5 was used as a control. GAPDH was used as an internal control.

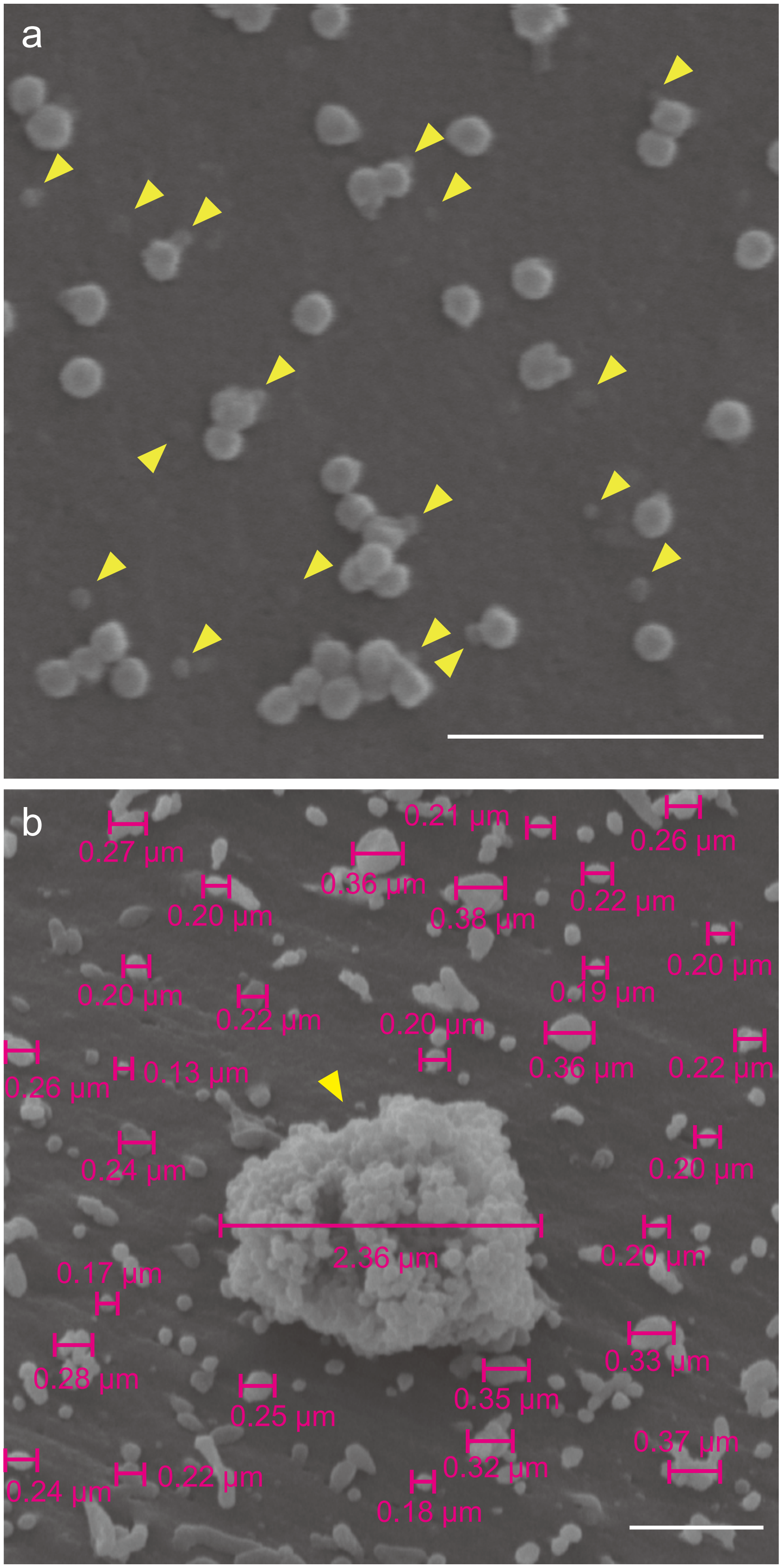

a
b
0.27 µm
0.21 µm
0.26 µm
0.36 µm
0.20 µm
0.38 µm
0.22 µm
0.20 µm
0.20 µm
0.19 µm
0.22 µm
0.20 µm
0.26 µm
0.13 µm
0.36 µm
0.22 µm
0.24 µm
0.20 µm
2.36 µm
0.20 µm
0.17 µm
0.28 µm
0.33 µm
0.25 µm
0.35 µm
0.37 µm
0.24 µm
0.22 µm
0.32 µm
0.18 µm

Supplementary Figure 4 | E-EVs shed from plasma membrane. Higher magnification of the SEM image showing that E-EVs are shed from the plasma membrane surface. (a) At 10 seconds after stimulation by CytoCombo+LPS of high dose. Smaller-sized (0.1 to 0.2 μm in diameter) of shedding (vesicles) can be observed. In addition, early phase shedding that is smallest-sized dome-shaped swellings (< 0.1 μm), emerges at the cell surface (arrowheads). (b) At 10 minutes after stimulation by CytoCombo+LPS of high dose. Many E-EVs shed from plasma membrane are ranging between 0.1 and 0.4 μm in diameter in b.End5 cells. Some of them aggregated to produce large size of E-EVs (up to 3 μm, arrowhead). Scale bar, 1 μm.

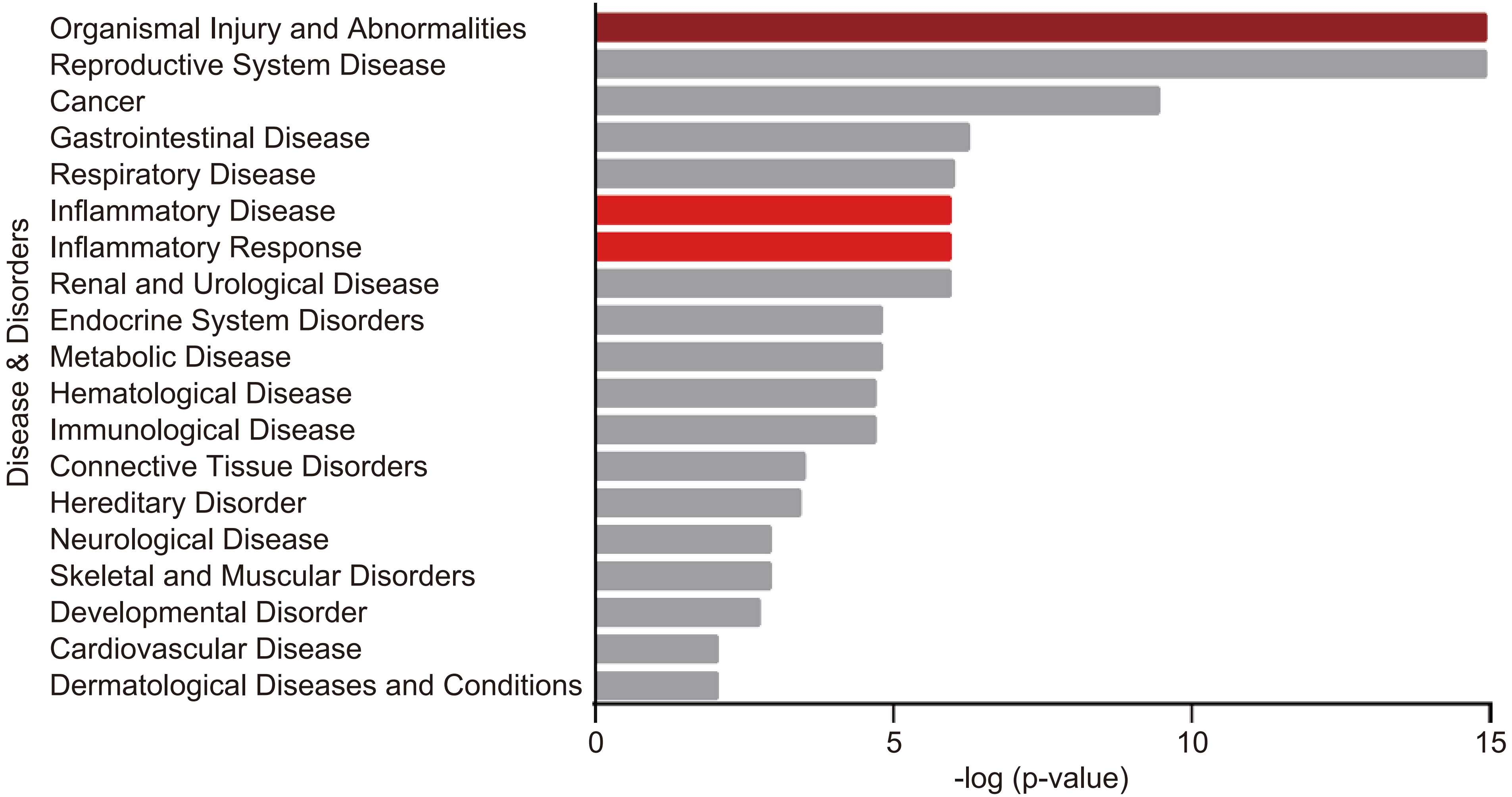
Organismal Injury and Abnormalities
Reproductive System Disease
Cancer
Gastrointestinal Disease
Respiratory Disease
Inflammatory Disease
Inflammatory Response
Renal and Urological Disease
Endocrine System Disorders
Metabolic Disease
Hematological Disease
Immunological Disease
Connective Tissue Disorders
Hereditary Disorder
Neurological Disease
Skeletal and Muscular Disorders
Developmental Disorder
Cardiovascular Disease
Dermatological Diseases and Conditions
Disease & Disorders
0
5
10
15
-log (p-value)

Supplementary Figure 5 | Inflammation-related miRNAs over-represent in inflammatory disease and responses. Inflammation-related miRNAs were classified by Ingenuity Pathway Analysis (IPA). Inflammatory Disease and Inflammatory Responses significantly over-represented in the miRNAs upregulated following stimulation of high dose CytoCombo+LPS are presented (bright red). Organismal Injury and Abnormalities, which may associate with inflammation, over-represented in the miRNAs upregulated following stimulation of high dose CytoCombo+LPS are presented (dark red).

Supplementary Table 1 | Concentration of the inflammatory stimuli.

Stimulant		0	Low	Middle	High
TNF-α		0 ng/ml	0.1 ng/ml	1 ng/ml	10 ng/ml
IL-1β		0 ng/ml	0.002 ng/ml	0.02 ng/ml	0.2 ng/ml
IFN-γ		0 ng/ml	0.1 ng/ml	1 ng/ml	10 ng/ml
LPS		0 μg/ml	2 μg/ml	20 μg/ml	200 μg/ml
CytoCombo	TNF-α:	0 ng/ml	0.1 ng/ml	1 ng/ml	10 ng/ml
	IL-1β:	0 ng/ml	0.002 ng/ml	0.02 ng/ml	0.2 ng/ml
	IFN-γ:	0 ng/ml	0.1 ng/ml	1 ng/ml	10 ng/ml
CytoCombo+LPS	TNF-α:	0 ng/ml	0.1 ng/ml	1 ng/ml	10 ng/ml
	IL-1β:	0 ng/ml	0.002 ng/ml	0.02 ng/ml	0.2 ng/ml
	IFN-γ:	0 ng/ml	0.1 ng/ml	1 ng/ml	10 ng/ml
	LPS:	0 μg/ml	2 μg/ml	20 μg/ml	200 μg/ml
